# Perspective on tailoring longitudinal structured beam and its applications

**DOI:** 10.1515/nanoph-2024-0720

**Published:** 2025-04-21

**Authors:** Alan E. Willner, Huibin Zhou, Xinzhou Su

**Affiliations:** Department of Electrical and Computer Engineering, 5116 University of Southern California, Los Angeles, CA 90089, USA

**Keywords:** structured light, Bessel beam, beam shaping, orbital angular momentum

## Abstract

Tailoring structured beams with varying lightwave properties along the longitudinal dimension has recently gained much interest. This paper gives a perspective on the advances of longitudinally structured beams, their potential applications, and future opportunities.

## 1 Prologue

The entire optics and photonics community is indebted to Prof. Federico Capasso for his profound and monumental impact on our field. He has dramatically influenced the research and applications areas worldwide, and we join everyone in wishing Prof. Capasso a very Happy 75th Birthday, and many more years of happiness, health, and impact.

This paper concerns the longitudinal structuring of light’s properties, and his vast contributions extend to our article. As one example, his pioneering work on metasurfaces for light transformation is seminal and is cited below.

## 2 Introduction

In general, a lightwave has several properties, including amplitude, phase, polarization, and wavelength. On its most basic level, varying the amplitude of light can be utilized for many important applications, such as communications, sensing, and imaging [1]. As such, there has been significant and growing interest in the field of “structured light”, in which multiple properties of light can be tailored in multiple spatial dimensions [1]. This subfield is also fairly young, since much of the activity began with the seminal 1992 paper (L. Allen, et al. [2]) that described how a beam of light that has a transverse vortex intensity spatial distribution and helical phasefront “twisting” during propagation can carry orbital-angular-momentum (OAM) [3], [4].

Any beam of light can be thought of as having “structure”, since any beam can be a summation of different spatial modes from a modal basis set [5]. Each mode from a set is orthogonal to all other modes, and each mode will have a unique complex coefficient and corresponding transverse phase, amplitude, and polarization. The summation of these modes can produce any arbitrary optical field in the transverse dimension, and any beam being composed of only a single mode is also considered “structured”. Examples of transverse structured light can be each spatial mode from the Laguerre Gaussian (LG_*ℓ*,*p*_) and Hermite Gaussian (HG_*m*,*n*_) modes [6]. In these modal sets, there exist two indices that define the transverse spatial distribution of each mode; for LG beams, *ℓ* describes the number of 2π phase changes in the azimuthal direction and *p* relates to the number of intensity rings [3].

There are various exciting applications of transverse structured light, including:

**(a) Communications**: Since each transverse spatial mode is orthogonal to other modes, communication systems can have dramatically higher capacity by two approaches:
***i. Multiplexing***: Multiple data-carrying beams each containing a single mode can be simultaneously transmitted in the same medium with little inherent channel crosstalk [7], [8], [9]. This is commonly referred to as mode-division-multiplexing (MDM), which can be considered a subset of space-division-multiplexing (SDM) [10].
***ii. Encoding***: Each symbol can be encoded on one of multiple orthogonal states, thus enabling many bits/symbol – which can be quite useful for photon-starved quantum links in which bits/photon is a critical metric [11], [12].
**(b) Sensing**: Due to light–matter interactions, power can be coupled from a well-defined amplitude and phase spatial distribution of a beam into other modes [13], [14]. Such transformation can be considered a “signature” that represents some property of a given medium or object [15].
**(c) Imaging**: The unique spatial distribution of structured light can be used in microscopy and imaging for increasing resolution [4]. For example, the spiral phase of OAM beams was used in phase contrast microscopy, demonstrating edge contrast enhancement [16].
**(d) Trapping**: The momentum can exchange between photons and microparticles, which produces a force for optical trapping [17]. Various transverse structured light has shown novel capabilities to the study of micromanipulation [18].

The above discussion relates to the tailoring of the transverse spatial properties of light all along the propagation. When neglecting divergence issues, this transverse structure is not designed at the transmitter to vary as it continues to propagate, e.g., a beam of a certain OAM value will stay that value as it propagates. However, light’s properties can also be designed at the transmitter to vary in the longitudinal dimension, such that some properties of light can appear at some distance and not appear at other distances [1].

One of the most basic and useful ways to longitudinally tailor a lightwave is a simple lens. The wave comes to a focus at a given distance. However, lenses are typically static, thus limiting the ability to arbitrarily tailor a beam longitudinally. Indeed, all of a lightwave’s properties can be designed to change at various propagation distances. A typical approach to longitudinally tailoring light is to judiciously utilize the longitudinal wavenumber, *k*_*z*_, which is typically thought of as part of the Bessel Gauss ( $BG_{\ell ,k_{z}}$ ) modal basis set [1], [19]. The two indices of BG beams are *ℓ* and *k*_*z*_, representing, respectively, the OAM value and the longitudinal propagation constant [20], [21]. Although there is much interest in BG beams due to their relatively lower beam divergence [20], such beams can also be used in uniquely tailoring the longitudinal properties of light.

One example of longitudinal structured light is where a beam composed of multiple BG modes of different *k*_*z*_ values is launched from a transmitter and yet can undergo various degrees of intensity focusing at different distances [22]. Changing the composite *k*_*z*_ values and their complex coefficients can change whatever longitudinal distribution that is desired [23], [24], [25]. This longitudinal structuring can be extended to multiple properties of light [26], [27], [28], [29], [30].

In this paper, we will discuss various advances in the ability to longitudinally structure light, potential applications of such an ability, and thoughts on specific exciting and future areas of research in this field.

## 3 Longitudinal structured beam (LSB)

Longitudinally structuring light can be thought of as *a priori* programming into a beam an ability such that it can morph into something unique at different distances. Importantly, this programming is governed by the selection and control of the longitudinal wavenumbers at the transmitter.

**Figure 1:**
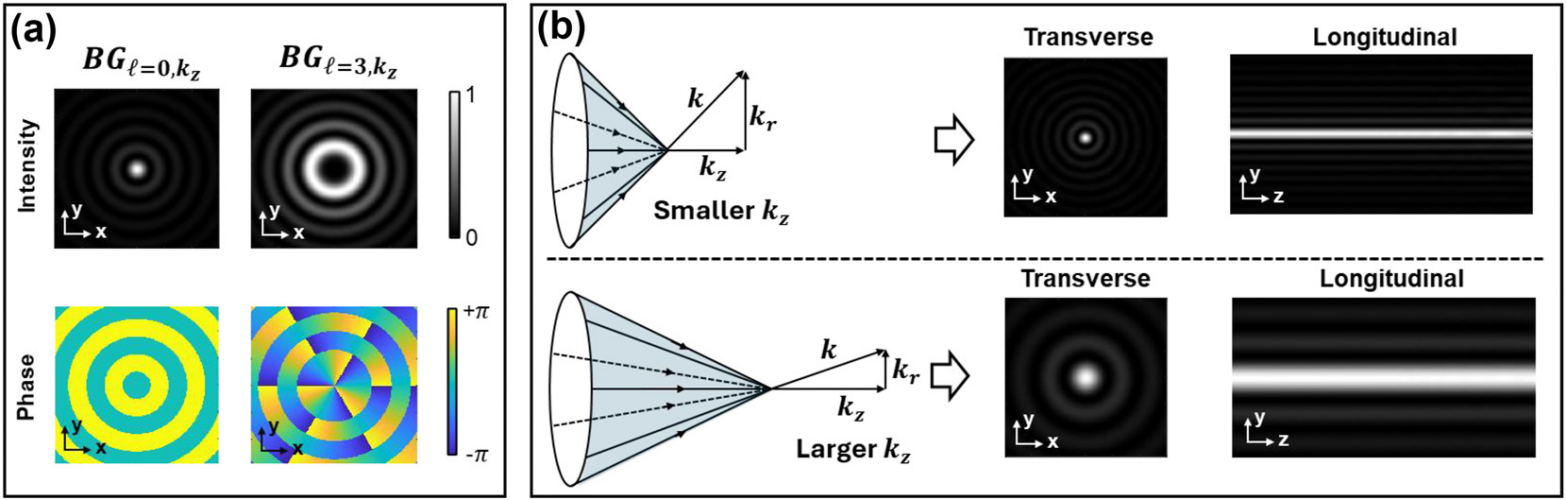
Transverse and longitudinal profiles for BG beams.** **(a) Transverse intensity and phase profiles for two examples of $BG_{\ell ,k_{z}}$ beams with different OAM values *ℓ* and the same *k*_*z*_. (b) Transverse and longitudinal intensity profiles for a $BG_{\ell =0,k_{z}}$ beam with different *k*_*z*_ values.

The longitudinal wavenumber (*k*_*z*_) of a lightwave generally refers to the component of the wavevector (*k*) that lies along the axial direction (typically the *z*-axis) [31]. For plane waves propagating in different directions, the *k*_*z*_ varies depending on the angle of propagation relative to the *z*-axis [31]. For a BG beam, the *k*_*z*_ is the longitudinal component of its wavevector $k=\frac{2\pi }{\lambda }$ , satisfying ${k_{r}}^{2}+{k_{z}}^{2}=k^{2}$ , where *k*_*r*_ represents the transverse wavenumber determining the radial structure of the BG beam (see Figure 1) [19], [32].

**Figure 2:**
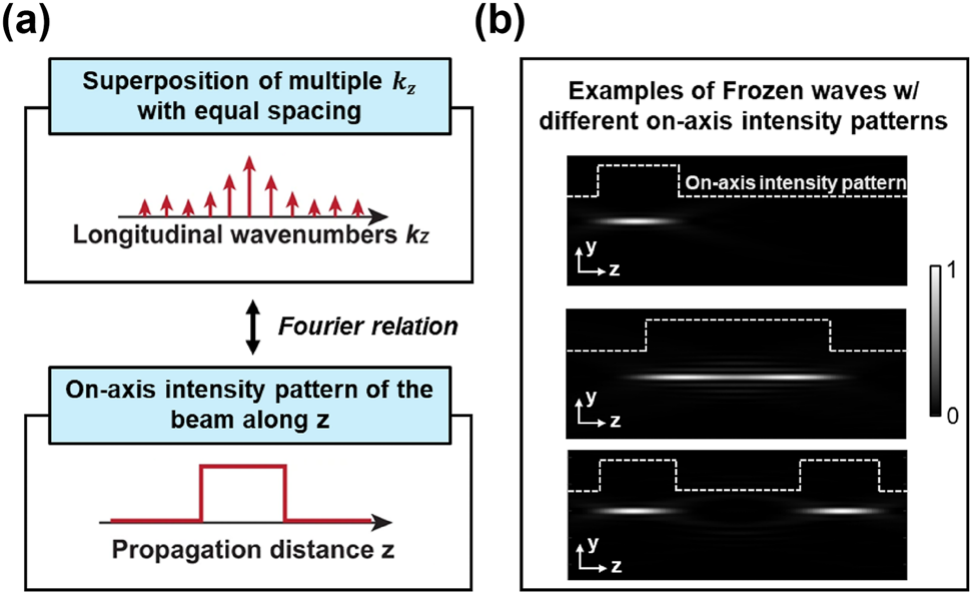
Tailoring LSBs by a coherent superposition of multiple BG beams carrying different *k*_*z*_. (a) Fourier relation between the longitudinal wavenumbers and the on-axis longitudinal intensity pattern. (b) Three examples of Frozen waves with different on-axis longitudinal intensity patterns.

**Figure 3:**
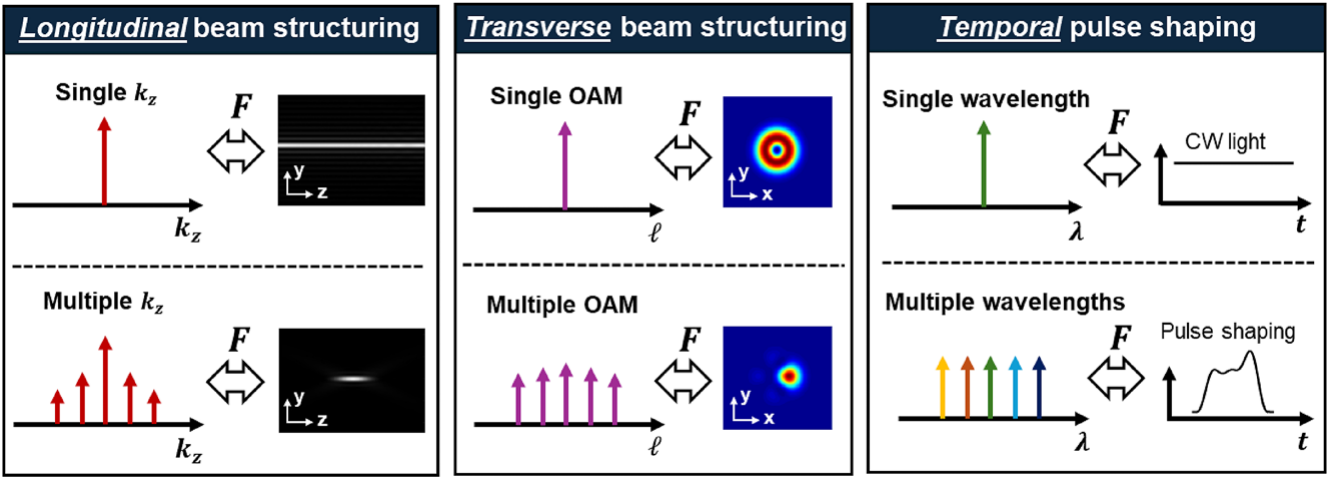
Longitudinal beam structuring is analogous to transverse beam structuring [34] by superposition of multiple transverse modes and temporal pulse shaping [37] by superposition of multiple wavelengths. ***F*** means Fourier transform. Transverse beam profiles are reprinted from Ref. [34], with permission from Optica Publishing Group. Temporal pulse shapes are reprinted from Ref. [37], with permission from Optica Publishing Group.

LSBs can be tailored by a coherent superposition of multiple BG beams carrying different *k*_*z*_ with equal spacing [22]. By designing the complex coefficients of each *k*_*z*_, one can control the constructive and destructive interference between different *k*_*z*_ components at different *z* distances. As a result, the beam has a higher on-axis intensity at some designed distances, while at other distances the on-axis intensity is low. Such on-axis intensity changes along the longitudinal direction form a longitudinal intensity pattern, which is determined by the complex coefficients of *k*_*z*_ through a Fourier transform (see Figure 2) [23], [25]. Such a beam is also named “Frozen wave” [22]. Indeed, as shown in Figure 3, this concept of longitudinal beam structuring is analogous to (a) transverse beam shaping by superposition of multiple transverse modes [33], [34], and (b) temporal pulse shaping by superposition of multiple wavelengths [35], [36].

**Figure 4:**
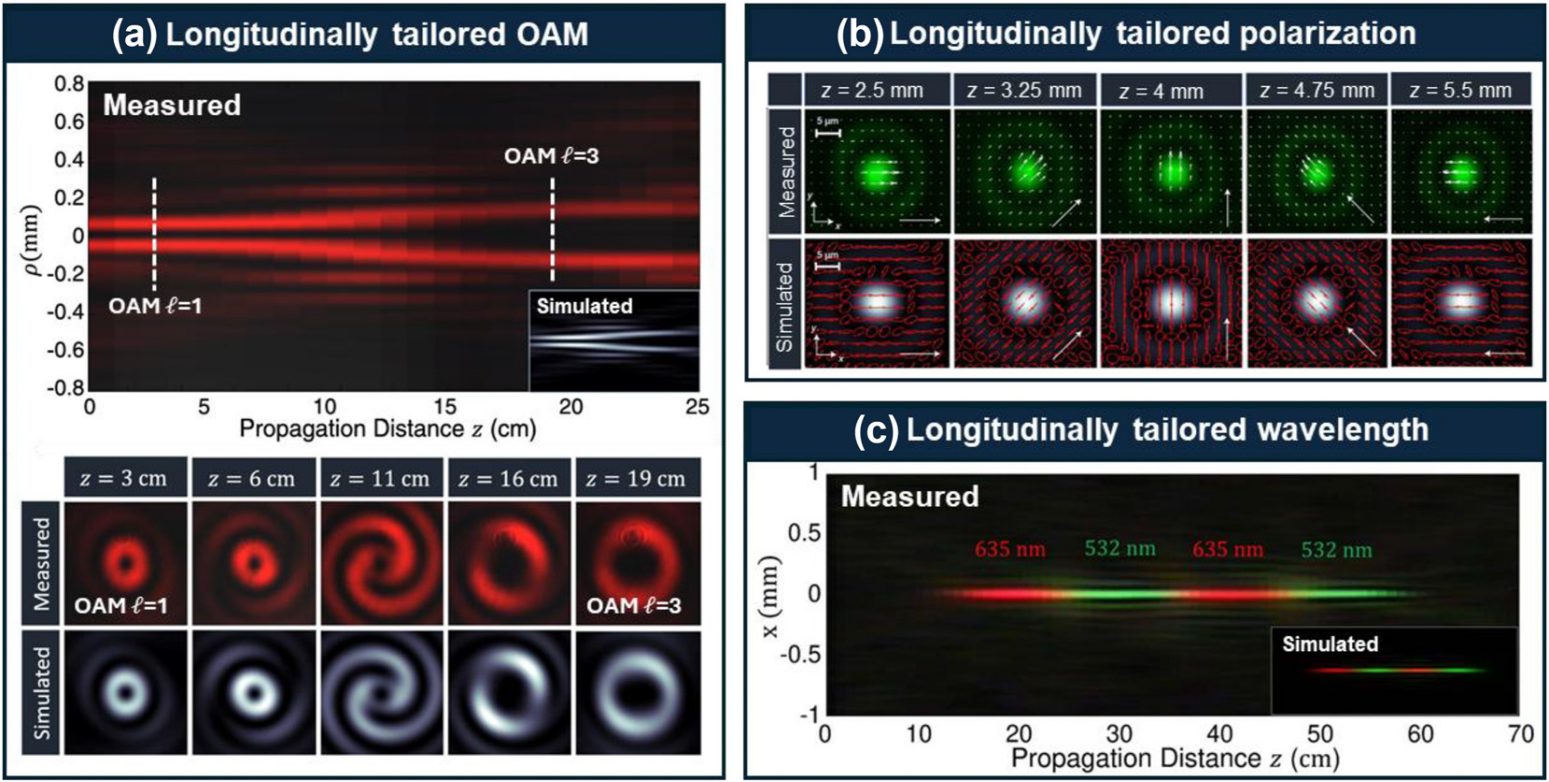
Examples of longitudinally tailored light properties, including (a) OAM [27]: OAM changes from *ℓ* = 1 to *ℓ* = 3 during the beam propagation, (b) polarization [28]: the white arrows indicate the polarization degrees at different distances, and (c) wavelength [29]: the wavelength oscillates between 635 nm and 532 nm along the propagation path. (a) is reprinted from Ref. [27], with permission from American Physical Society. (b) is reprinted from Ref. [28], with permission from American Physical Society. (c) is reprinted from Ref. [29], with permission from Optica Publishing Group.

Besides intensity, longitudinal controls of several other on-axis light properties have also been demonstrated, including OAM, polarization, and wavelength. Figure 4 shows some examples, in which their generation share a similar mechanism; that is, multiple Frozen waves each carrying a different light property are superposed such that specific properties can appear at designed distances [27], [28], [29].

There are several issues one should consider when designing and generating LSBs:

**(a) Transmitter (Tx) aperture size**: For a single BG beam with a given *k*_*z*_, the size of the Tx aperture limits its non-diffraction propagation region [22]. Moreover, a smaller *k*_*z*_ has a smaller non-diffraction region for a given Tx aperture size. Therefore, the maximum working distance of a LSB composed by multiple BG beams is limited by both the Tx aperture size and the minimal *k*_*z*_ value [22].
**(b) The spacing between** ***k***_***z***_: Based on the Fourier relation between the designed longitudinal pattern and *k*_*z*_, the spacing between neighboring *k*_*z*_ is determined to be 2*π*/*L*, where *L* is the maximum distance range designed for the LSB [22].
**(c) The number of** ***k***_***z***_: By utilizing more *k*_*z*_, one can generate an LSB with a more accurate longitudinal pattern as designed [23]. However, the number of *k*_*z*_ is limited by several factors. Specifically, according to above mentioned (a) and (b), the lower limit and the spacing of *k*_*z*_ values are determined by the Tx aperture size and the maximum working distance *L*. Moreover, the upper limit of *k*_*z*_ is *k* = 2π/λ [22].

Various approaches have been reported for the generation of LSBs. One approach involves utilizing a bulky spatial light modulator (SLM) [24], [30], which implements a computer-generated hologram to spatially modulate the complex amplitude of a light field at z = 0. This light field then propagates and exhibits longitudinally varying properties at different distances. By modifying the holographic pattern of the SLM, the LSB can be tuned to achieve different longitudinal properties [24], [30]. Recently, a more compact approach using metasurfaces has been demonstrated for generating LSBs [38]. Such a metasurface is a thin, passive device with a specifically designed distribution of subwavelength structures, acting as a hologram to tailor an input light field for LSB generation [38]. Notably, the transformation function of a metasurface can be designed to depend on the input light field [38]. For example, the metasurface can switch between different holograms by altering the input light’s incidence angle, polarization, or spatial mode [38], [39]. Therefore, the same metasurface can be used to generate different LSBs by modifying the input light field [39].

## 4 What can we do with the LSB?

**Figure 5:**
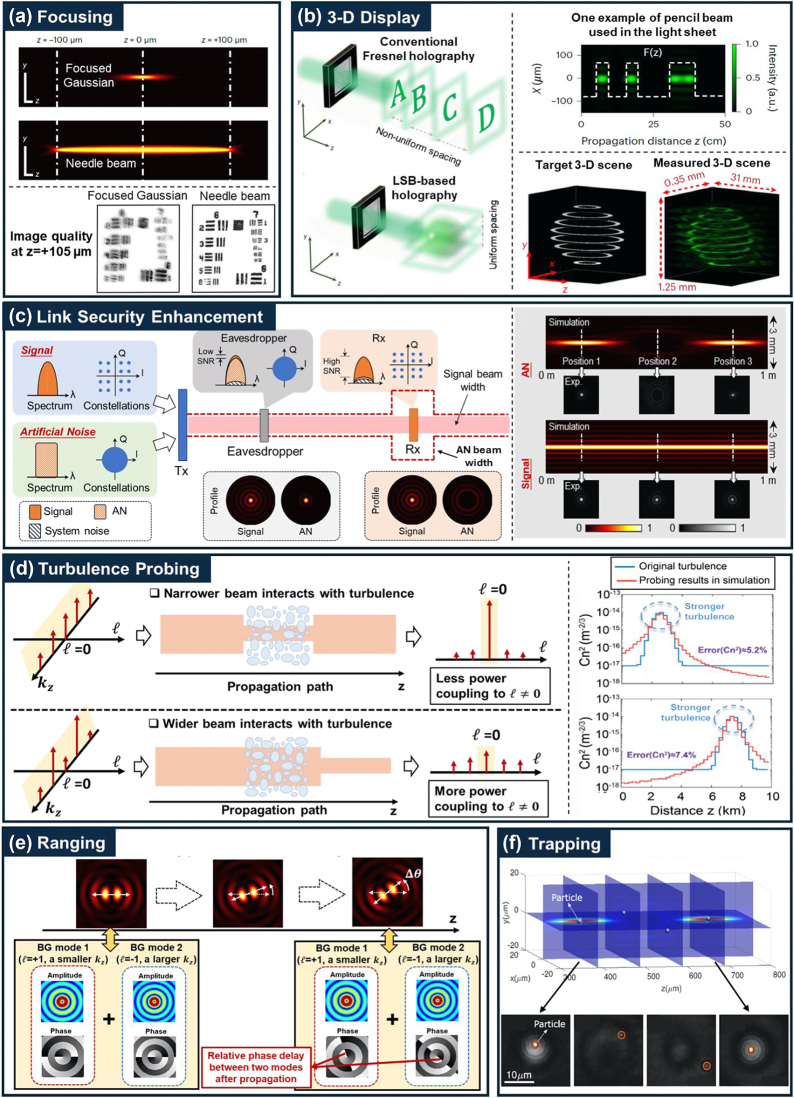
Various applications of LSBs: (a) Focusing [40]: a longitudinally structured needle beam was used to enhance depth of field and image resolution in optical microscopy. (b) 3-D display [41]: LSB-based holography was used for uniform reconstruction of 3-D objects with high resolution and low crosstalk. (c) Link security enhancement [43]: a longitudinally structured artificial-noise (AN) carrying beam was transmitted to produce low SNR at all distances except for where it is tailored to be much wider and thus not overlapping with the data beam. (d) Turbulence probing [46]: multiple longitudinally structured probe beams were tailored to have distance-dependent beam width in order to probe the distribution of turbulence strength along a path. (e) Ranging [49]: a longitudinally structured ranging beam has a petal-like transverse intensity profile that rotates along the beam propagation. (f) Trapping [56]: LSB was used to enable longitudinal trapping of microparticles in multiple transverse planes. (a) is reprinted from Ref. [40], with permission from Springer Nature Group. (b) is reprinted from Ref. [41], with permission from Springer Nature Group. (c) is reprinted from Ref. [43], with permission from Optica Publishing Group. (d) is reprinted from Ref. [44], with permission from Springer Nature Group. (e) is reprinted from Ref. [49], with permission from Elsevier. (f) is reprinted from Ref. [56], with permission from Optica Publishing Group.

The advances described above in the longitudinal structuring of light can potentially enable a number of important applications. The following list shows some recent examples of utilizing longitudinal structuring:

**(a) Focusing**: Achieving high-resolution optical imaging typically requires a tightly focused optical beam to attain diffraction-limited resolution in optical microscopy. However, the conventional focused beam results in a shallow depth of field (DOF), leading to reduced longitudinal resolution when moving away from the optical focal plane [40]. A longitudinally structured “needle-shaped” beam has been demonstrated to extend the DOF (see Figure 5(a)) [40]. Such a needle beam is generated by customized diffractive optical elements, which shape the transverse spatial phase profile of an input Gaussian beam and generate many closely adjacent foci along the longitudinal direction. Experimental results show the needle beam has a DOF up to a 28-fold Rayleigh length (14× longer than a focused Gaussian beam) [40].
**(b) 3-D Display**: Creating high-quality 3D scenes through computer-generated holography is highly important in many applications, such as AR and VR. Traditionally, 3D objects are reconstructed from a single hologram by projecting multiple 2D transverse scenes along and perpendicular to the optical path (i.e., longitudinal direction). However, in this approach, the spatial separation of these 2D scenes is fundamentally limited by the hologram’s numerical aperture, restricting the longitudinal resolution and depth perception of the generated 3D display [41]. To address this issue, a novel class of hologram has been recently demonstrated (see Figure 5(b)) [41], in which desired scenes are projected onto multiple 2D light sheets that are perpendicular to the display screen (instead of the optical path). Each 2D light sheet consists of an array of many longitudinally structured beams (also called “pencil-like” beams in this work) with intensity envelopes that can be locally structured along the propagation direction as needed. This approach can achieve continuous and uniform reconstruction of objects along the optical path with high axial resolution and low crosstalk [41].
**(c) Link Security Enhancement**: Securing data transmission against eavesdropping is a key requirement in communication systems [42]. FSO links are considered to be physically easy to intercept if an eavesdropper is in a data beam’s line of sight. LSB has been demonstrated to enhance the physical layer security of FSO links and prevent eavesdropping at unintended line-of-sight locations (see Figure 5(c)) [43]. Specifically, a data-carrying beam and a longitudinally structured artificial-noise-carrying beam can be transmitted together. The artificial noise (AN) beam can be made to narrow or widen at any given distance region by adjusting the complex coefficients of *k*_*z*_ components. Therefore, it can be designed to produce low signal-to-noise ratio at all distances except for where it is tailored to be much wider and thus not overlapping with the data beam. As a result, the data would only be readily detected at the location of a legitimate receiver.
**(d) Turbulence Probing**: Atmospheric turbulence can cause significant issues in various applications, including (i) injuring aircraft and airline passengers [44], and (ii) degrading free-space communication and imaging systems [45]. Understanding the inhomogeneous spatial distribution of turbulence is critical to help avoid and mitigate turbulence. LSB has been demonstrated to probe the strength distribution of turbulence along a propagation path (see Figure 5(d)) [46]. Due to light–matter interactions, a beam can couple power from one OAM mode into other OAM modes [47]. This power coupling depends on the beam size, such that a narrower beam has less interaction with the turbulence than a wider beam [47]. In this turbulence probing approach, multiple longitudinally structured beams are transmitted sequentially, each being tailored to be a superposition of multiple *ℓ* = 0 order BG modes with different *k*_*z*_. By controlling the complex coefficients of *k*_*z*_, the beam size of each beam can be designed to be distance-dependent, resulting in different distance- and turbulence-dependent modal coupling from the OAM *ℓ* = 0 order to other orders [46]. Based on measured modal coupling, the inhomogeneously distributed turbulence strength along the propagation path can be extracted.
**(e) Ranging**: There is growing interest in optical ranging in underwater environments [48]. A typical approach is based on measuring the time-of-flight (ToF) of a transmitted pulse reflected by a target object. However, underwater scattering can cause temporal spreading of the pulse and degrade ToF ranging accuracy [48]. Alternatively, an LSB-based ranging approach (see Figure 5(e)) has been shown relatively less sensitivity to scattering [49], [50]. Specifically, a CW beam consisting of two BG modes with different OAM orders and *k*_*z*_ is transmitted and reflected by an object. The interference between the two OAM orders results in a transverse petal-like intensity profile [32], [49]. The two *k*_*z*_ causes a distance-dependent phase delay between the two modes, resulting in rotation of the petals as the beam propagates [32], [49], [51]. Therefore, the object’s location can be detected by measuring the angle rotation of the petals. Moreover, the performance of this approach has been recently improved by utilizing more *k*_*z*_ to (i) increase the power density of the petals [52], and (ii) enable an attenuation-resilient property of the petals when propagating in turbid water [25], [53]. Additionally, the concept of rotating petals has also been demonstrated for measuring an object’s longitudinal velocity [54] and the refractive index of a medium [32], [55].
**(f) Trapping**: The techniques of optical trapping have become an important tool in various research fields and applications. It has been shown that the OAM beam can trap particles and drive them to move around the beam’s phase singularity in a transverse plane [18]. Recently, reports have shown that it is also possible to enable longitudinal trapping of microparticles in multiple transverse planes using longitudinal structured beams (see Figure 5(f)) [56], [57]. A longitudinally structured beam with high central intensity in two separate distance regions was used for trapping. The distribution of the optical force field around the center of the two regions was investigated. Experimental results show the microparticles can be trapped in different transverse planes located at the center of the two regions [56].

## 5 Future research directions

**Figure 6:**
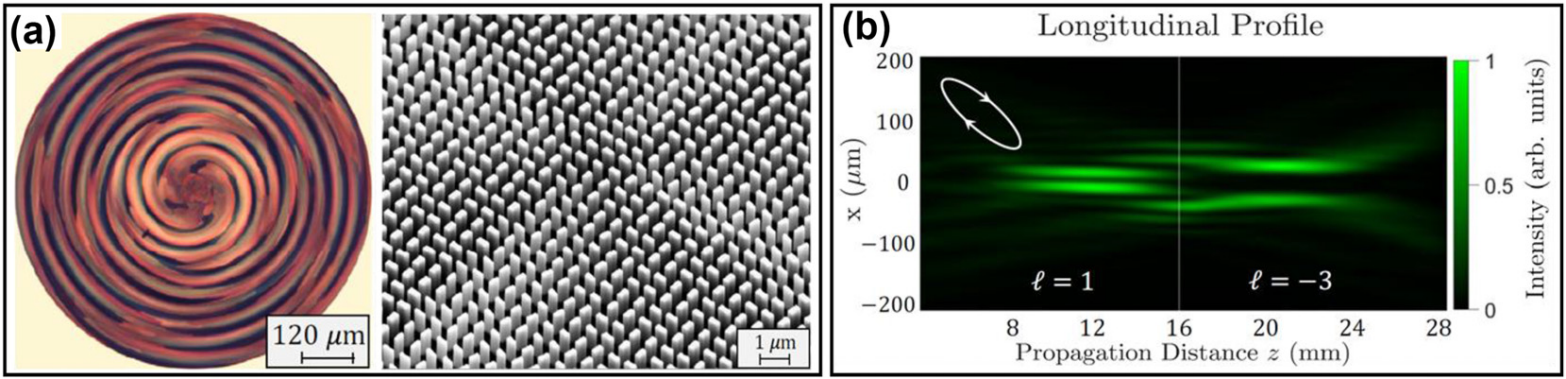
Metasurface for the generation of LSBs. (a) Metasurface designed and fabricated by Prof. Capasso’s group for generating LSBs [60]. (b) Measured LSB with longitudinally tailored OAM generated by such metasurface [60]. (a) and (b) are reprinted from Ref. [60], with permission from Springer Nature Publishing Group.

**Figure 7:**
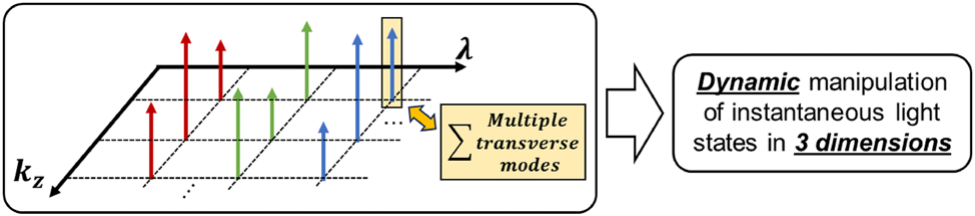
Simultaneous tailoring of the beam in transverse, longitudinal, and wavelength domains can potentially enable dynamic manipulation of instantaneous light states in 3 dimensions.

The subfield of longitudinal structuring of light is still fairly young, with many of the early seminal papers dating back only to the early 2000’s [22], [23]. The amount of research in this area and interest in advancement has been growing, and the future promises to be rich in exciting research topics to explore. These range from a basic understanding of wave propagation to device technologies for tunable longitudinal beam control. Some examples of future research directions include the following:

**(a) Integration**: Reducing size, weight, and power (SWaP) is crucial for generating LSBs in practical applications. Two promising approaches are: (a) Photonic Integrated Circuits (PICs): Integrating multiple antennas on a single chip enables precise control of Bessel beam generation [58], [59], though complex longitudinal tailoring remains underexplored. (b) Metasurfaces: Ultra-thin structures with high-resolution subwavelength elements manipulate light properties, offering longitudinal control of multiple parameters (see Figure 6) [39], [60]. However, their tunability requires further investigation.
**(b) Multi-dimensional**: As previously mentioned, longitudinal spatial control is analogous to temporal and transverse spatial control, as all are fundamentally based on the Fourier relationship between the spatial, temporal, or longitudinal domains and their respective wavelength domains. The tailoring of these dimensions is inherently compatible, allowing for precise manipulation across multiple domains [37]. By integrating multi-dimensional control, it becomes possible to achieve dynamic manipulation of instantaneous light states *(e.g., longitudinal-varying spatiotemporal structured light* [37], [61]*)* (see Figure 7)
**(c) Quantum**: An important basic understanding was the idea that a single photon can be transversely structured and carry OAM [62], similar to a classical optical beam. An interesting area of exploration might be determining the similarities and differences between a photon and a classical beam when it comes to longitudinal structuring *(e.g., z-dependent entanglement in quantum systems and in classical light fields* [63]*)*. Moreover, it could be quite interesting to study potential applications for quantum systems when longitudinally structuring a single photon.
**(d) Fiber**: Most of the longitudinal structuring of light has been demonstrated for free-space propagation, in which light can be tailored to exist in almost any mode or wavenumber. On the other hand, a waveguide (e.g., fiber) has inherent constraints on the allowable mode and wavenumber combinations. An interesting scientific exploration could be the control, limitations, and applications of the propagation of longitudinally structured light in fiber and integrated waveguides [64].
**(e) Wavelengths**: Although this paper has dealt primarily with visible and near-infrared optical wavelengths, it can be assumed that the fundamental physics would likely hold true for many other regions of the electromagnetic (EM) spectrum; this was shown to be true for transverse structured EM waves [65], [66], [67], [68], [69]. However, the specific engineering implementations, systems limitations, and potential applications might be quite different for wavelengths outside of the above regions.
**(f) Nonlinear interaction**: In Section 4, we discuss several example applications of LSBs in the linear regime of light–matter interactions. LSBs may also offer new opportunities for research on nonlinear interactions. For instance, LSBs can be designed with arbitrary beam-focusing regions distributed along the propagation path, enabling control over the beam’s spatial power density at different distances [22]. This z-dependent power density may help to explore various nonlinear light–matter interactions in the longitudinal dimension [70]. Additionally, it could also be interesting to study the generation and tailoring of different LSBs through nonlinear wave mixing [61].

## 6 Summary

Tailoring multiple properties of light longitudinally is a great complement to tailoring light transversely, such that there is a toolkit for producing almost any kind of light at any given location. Although it is unclear what applications will ultimately be impacted by this capability, there is much excitement in finding out what is possible. Indeed, we would venture to say that there is much confidence that such unique capabilities will be used in ways we may not yet envision.
